# Umbilical two-port laparoscopic percutaneous extraperitoneal closure for patent processus vaginalis in boys: incision-hiding and solo-like surgery

**DOI:** 10.1186/s12893-021-01277-1

**Published:** 2021-06-02

**Authors:** Yuanhong Xiao, Zhou Shen

**Affiliations:** Department of Pediatric Surgery, Faculty of Pediatrics, the Seventh Medical Center, Chinese PLA General Hospital, Nan Men Cang 5th, Dongcheng District, Beijing, 100700 China

**Keywords:** Boys, Child, Laparoscopy, Minimal invasive surgery, Patent processus vaginalis closure

## Abstract

**Background:**

Transumbilical two-port laparoscopic percutaneous extraperitoneal closure for the treatment of processus vaginalis patency in boys has been practising recent years. The applicable instruments and skills are still evolving. In this study, we used a self-made needle assisted by a disposable dissecting forceps to practise this minimal invasive method for patent processus vaginalis in boys. Its safety and effectiveness were studied. The methods for depth and orientation perceptions were analyzed.

**Methods:**

From January 2020 to November 2020, boys characteristic of symtomatic patency of processus vaginalis were performed open surgery consecutively. From December 2020, the authors begun to propose transumbilical two-port laparoscopic percutaneous extraperitoneal closure for this kind of boy patients. The open group included fifteen boys and the laparoscopic group included ten ones. The data of the patients age, constituent ratios of unilateral and bilateral patency, operating time, postoperative stay in hospital, follow-up time, conversion, postoperative complications were assessed. Throughout the laparoscopic process, the parallel and synchronous movements of lens pole and dissecting forceps were maintained. Vas deferens protrude was imagined as one of the point to form the triangular manipulation plane.

**Results:**

There were no statistically significant difference between the laparoscopic group and the open group for the following items: age, operating time, the constituent ratios of unilateral or bilateral patency of processus vaginalis (P > 0.05). Postoperative stay in hospital and follow-up time of the laparoscopic group was significantly shorter than that of the open group (P = 0.0000). No laparoscopic case was converted to open surgery. After 10 cases of laparoscopic practice, orientation perception was established. There were no postoperative complications for all the patients.

**Conclusion:**

Our preliminary experience suggested that umbilical two-port laparoscopic percutaneous extraperitoneal closure is safe and convenient for patent processus vaginalis treatment in boys. It has the advantage of incision-hiding and can be manipulated like a solo-like surgery.

## Background

Congenital patent processus vaginalis in hernia or hydrocele was one of the most commonly encountered circumstances for male children. The symptomatic incidence of this disease was 1/150. Open surgery through inguinal canal approach was the traditional manipulation for this disease. Since 2006, laparoscopic intraperitoneal or percutaneous extraperitoneal internal ring closures have been reported [1–4]. Recently, laparoscopic percutaneous extraperitoneal closure of internal ring has been increasingly emerged [5–11]. The concept of single-port laparoscopic percutaneous extraperitoneal closure of the patent processus vaginalis was adopted for its obvious advantages of incision-hiding and simplicity [12]. Another merit was its space-sparing without more instruments intervention in the limted abdominal cavity of children. However, this simplified laparoscopic method was more fit for experienced surgeons. For the novices, they not only faced with depth perception difficulty, but also eye-hand coordination difficulty. And double-hand manipulation was beneficial for depth perception and orientation perception, which were the cornerstones for laparoscopic surgery [13, 14]. Umbilical one site two-port laparoscopic method achieved the same cosmetic effect with that of the single-port laparoscopic one [15, 16]. Obviously, how the two-port instruments manipulating simultaneously in the limited umbilical area was the key point for the technique performance. This showed that more details of technical improvements were still deserved of being studied for the purpose of shortening the learning curve of this advanced technology. In our study, we practiced the technique through umbilical two-port laparascopic percutaneous extraperitoneal closure for patent processus vaginalis in boys. The solo-like surgical manipulation of lens pole and assissting forceps and the orientation perception of this technique were analysed.

## Methods

### Population and data collection

From December 2020, the authors begun to perform umbilical two-port laparoscopic percutaneous extraperitoneal closure for the boys who suffered from symptomatic patent processus vaginalis (hydrocele or hernia). We also retrospectively studied the patients who were consecutively accepted traditional open inguinal procedures from January 2020 to November 2020. The laparoscopic group included 10 boys with 13 patent processus vaginalis. The open group included 15 boys with 17 patent processus vaginalis (Table 1). The data of age, operating time, postoperative time in hospital, follow-up time were assessed (Table 2). There was no conversion of laparoscopic surgery to an opened one. All the patients had no postoperative complications. All the parents signed the informed contents for open or laparoscopic surgery. And all the parents signed the informed contents to participate in this study. We confirmed that all methods were performed in accordance with the relevant guidelines and regulations.

**Table 1:** Patient characteristics

Items	Open group	Laparoscopic group	P value
Age (year)	3.677 ± 2.327 (1.83–10)	5.409 ± 3.283 (1.5–12)	0.1694
Cases	15	10	
Sides	17	13	
Left	4	3	
Right	9	4	
Bilateral	2	3	0.3577

**Table 2:** Patient outcomes

Items	Open group	Laparoscopic group	P value
Operating time (min)	57.333 ± 20.208 (35–108)	51.000 ± 18.227 (30–80)	0.4244
Postoperative time (days)	3.000 ± 0.854 (1–5)	1.300 ± 0.483 (1–2)	0.0000
Follow-up time (m)	6.067 ± 1.831 (3–9)	1.000 ± 0.667 (0–2)	0.0000

### Surgical processes

**Fig. 1:**
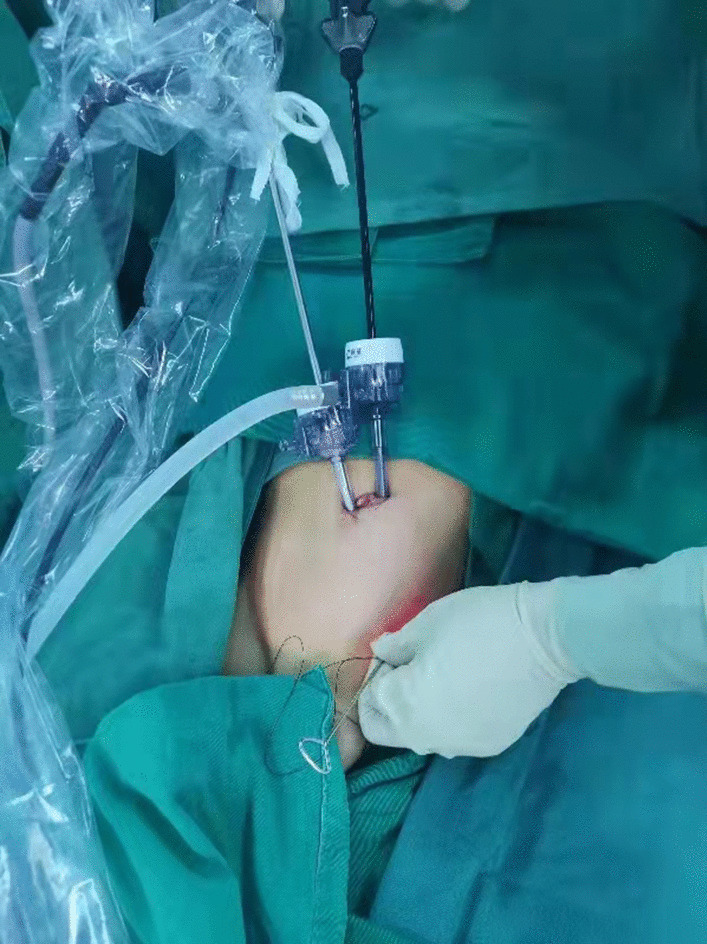
Umbilical two-port assignment of the dissecting forceps and the lens. The operator stood at the left side of the patient with left hand handing the needle and right hand controlling the forceps. The lens pole maintained parallelly and synchronously with the forceps pole

Patient lay in the supine position under general anesthesia. For open repair, inguinal transverse incision was adopted. The inguinal canal was opened. Patent processus vaginalis was dissected and seperated from the spermatic cord. High ligation of the patent processus vaginalis at the level of internal ring was performed with silk thread. For laparoscopic operation, the surgeon stood at the left side of the patient. The assistant stood at the right side. The screen was placed at the patient feet side. A 5 mm arc incision along the right verge of the umbilicus was made first. After blunt dissection of the subcutaneous tissue with a mosquito clamp, the operator and assistant lifted the lateral abdominal wall of the incision respectively, and the operator inserted a 5 mm disposable Trocar through the incision into the abdominal cavity. Then the lens (5 mm, 30° optics) was introduced from the Trocar to the cavity. Carbon dioxide was poured into the cavity with a pressure of 8–12 mmHg. When the abdominal cavity was distended, the second incision oppostie to the first one was made on the left verge of the umbilicus. Another 5 mm disposable Trocar was inserted through this incision and a disposable dissecting forceps (30-degree bend, Hunan Handlike Minimally Invasive Surgery CO., Ltd) was inserted through this Trocar into the abdominal cavity (Fig. 1). Peritoneal cavity was inspected first to preclude accidental injuries of the abdominal wall and viscera. The affected side was re-confirmed, and the opposite side was simultaneously assessed. For the asymptomatic patent processus vaginalis found during operation, internal ring ligation was performed simultaneously.

**Fig. 2:**
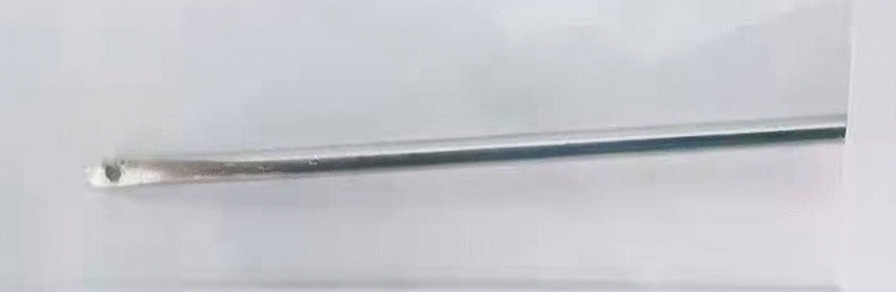
The self-made needle with a round dull end and a central hole. The silk thread could be passed through this hole

**Fig. 3:**
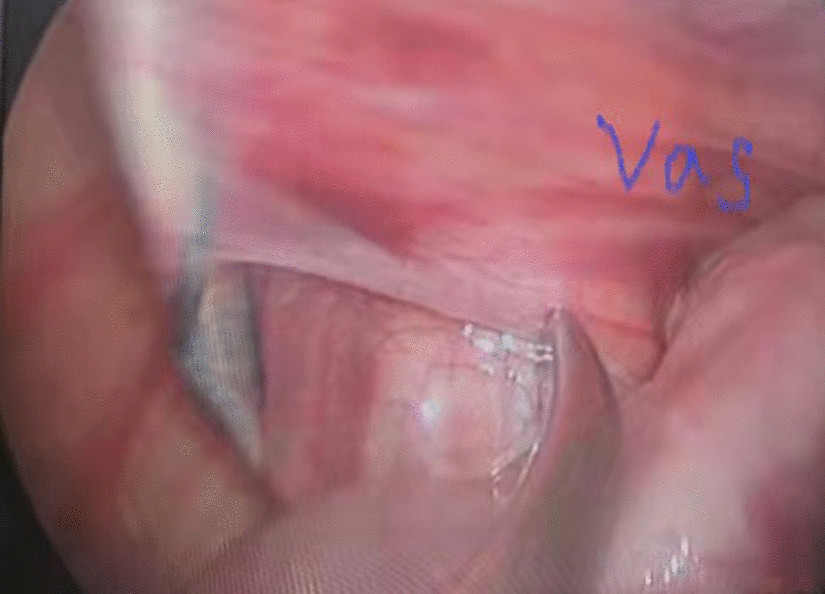
The needle had passed through vas deferens and vessels preperitoneally

**Fig. 4:**
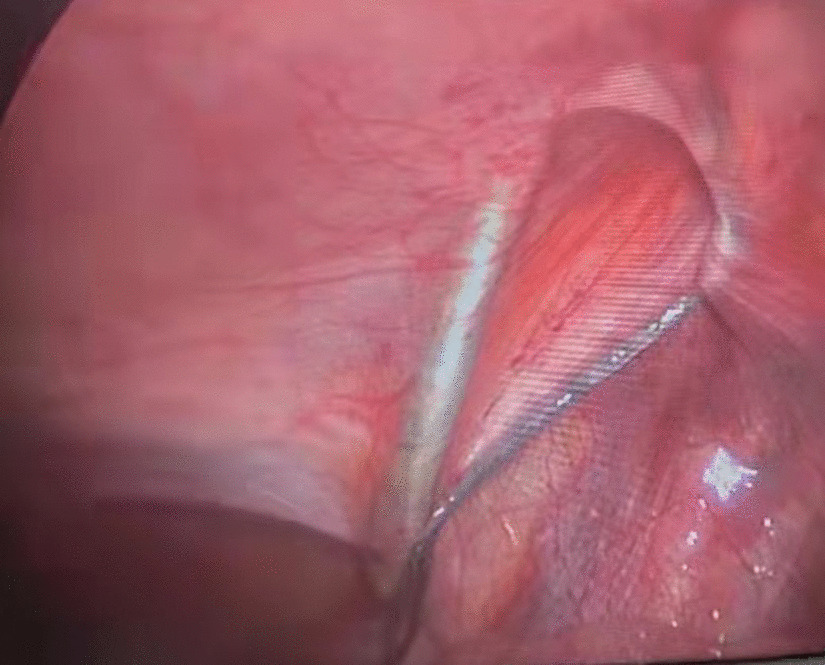
The thread loop had been maintained in the abdominal cavity for capturing the coming second thread loop, which was in the needle piercing the opposite semi-circle of the internal ring

**Fig. 5:**
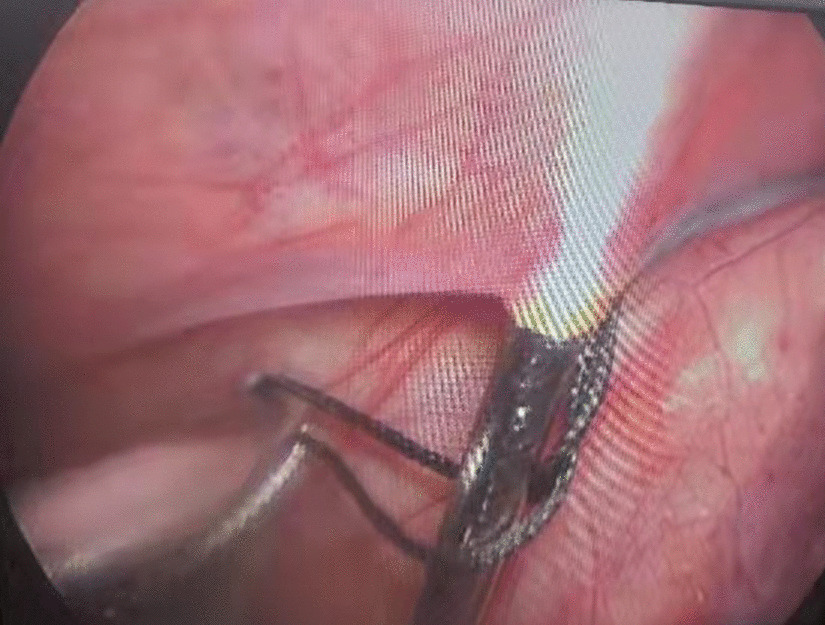
The needle with the second loop in its end hole passing through the first thread loop

**Fig. 6:**
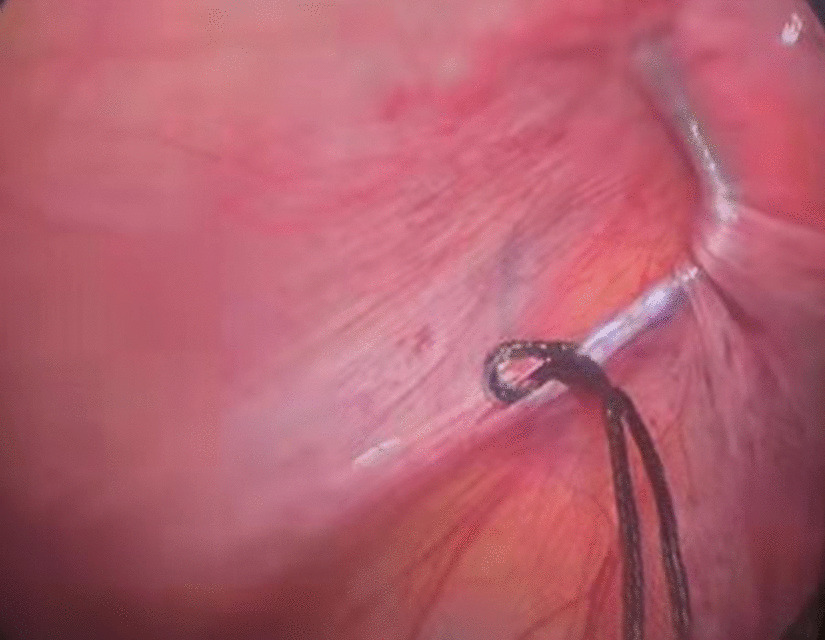
The second thread loop had been captured by the first thread loop

**Fig. 7:**
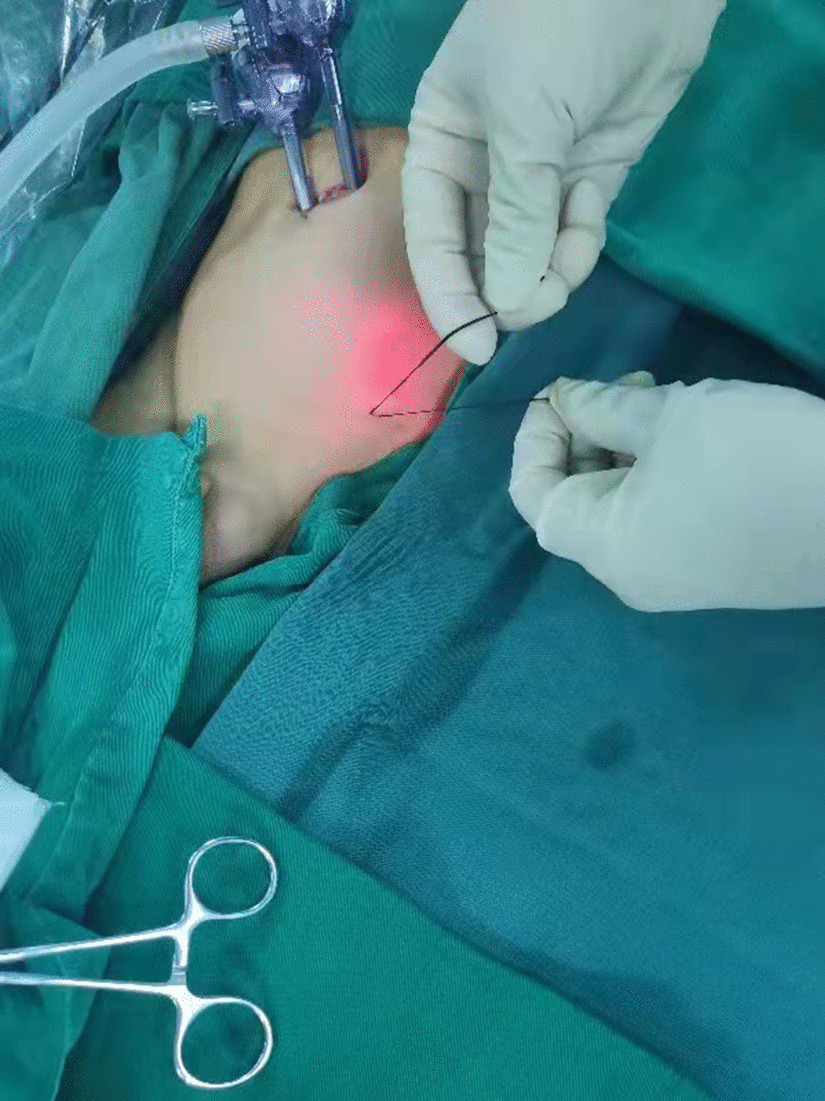
The second thread loop had been pulled out and circled the internal ring completely and circularly. And it was ligated extracorporeally

**Fig. 8:**
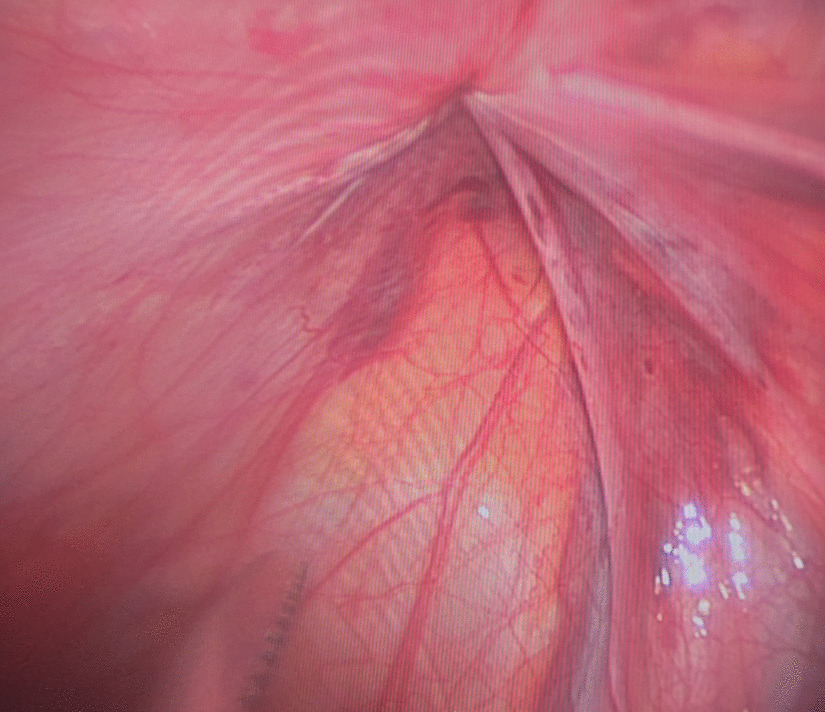
The laparocopic inside view of the internal ring closure

**Fig. 9:**
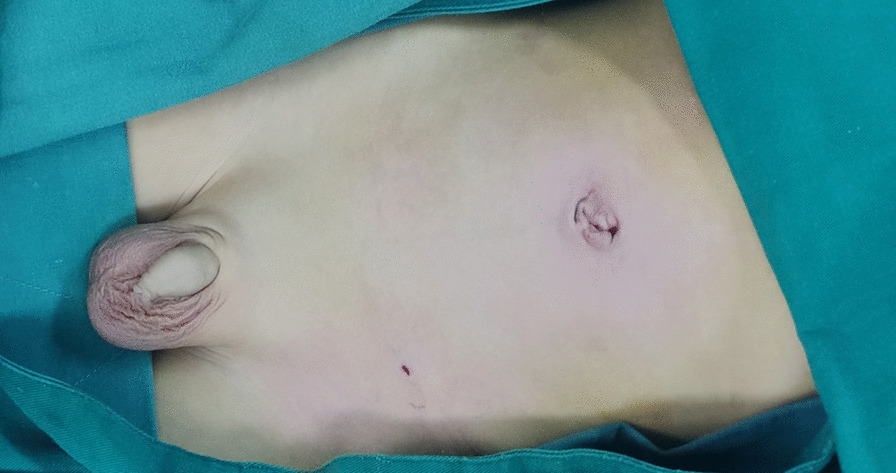
The umbilical two-port incisions and the affected groin incision

Then a tiny incision of 2 mm was made on the affected groin side just above the level of the internal ring. A shovel-like self-made needle with a central hole was used (Fig. 2). A silk thread was inserted through the hole beforehand. The operator held the needle with one hand with abdominal wall lifting with the other hand. The needle was pierced into the preperitoneal space through the groin tiny incision. Then the operator held the needle with left hand and dissecting forceps with right hand, observing the operative field under the lens controlled by the assistant. The len pole and the assisting forceps was always maintained parallelly and synchronously during the whole process. Then the needle moved preperitoneally towards the vas deferens site firstly. With the dissecting forceps assisting, the needle separated the peritoneum from the vas deferens. After the vas was passed, the vessels were passed without much more difficulty (Fig. 3). After the vessels were seperated from the peritoneum, the needle pierced into the peritoneal cavity. With the thread loop holding by the assisting forceps, the needle retreated outside of the body with the residual portion of the thread staying outside of the abdominal wall. The thread loop was remained in the cavity temporarily (Fig. 4). Once more, another silk thread was inserted in the central hole of the needle. Again, the needle with the thread loop was pierced into the preperitoneal space through the same groin incision and advanced through the opposite semi-circle of the internal ring. When the needle end reached the peritoneal puncture point where the first thread loop was passed through, the needle passed through the point and was continuously introduced into the loop for a certain length (Fig. 5). With the dissecting forceps holding the second thread loop, the needle was withdrawn out of the body. Similarly, the residual portion of the second thread was also satying outside of the abdominal wall. Then the first loop was pulling out of the body to bring the second thread loop out simultaneously (Fig. 6). Then the operator took the two ends of the second loop and tied the two strands together to complete the total closure of the internal ring preperitoneally (Figs. 7, 8). The knot was buried under the groin skin. After the carbon dioxide was released from the abdominal cavity, the umbilical two trocars were pulled out. The incisions were closed subcutaneously with 5–0 absorbable thread, and the skin verge was faced and adhered with medical adhension agent (Fig. 9). The incisions were covered with waterproof dressings. For the patients with hydrocele, the liquid was sucked out by a syringe needle from the scrotum. During the whole procedure, extreme care was taken not to damage the inferior epigastric, femoral and external iliac vessels.

### Data analysis

Data analysis was performed using CHISS (Chinese High Intellectualized Statistical Software) software version 2004. Student T Test was used to compare the distribution of quantitative variables between groups (P65-68, Medical Statistics and CHISS Application) [17]. Pearson Chi-square Test was used to compare the distribution of the qualitative variables between groups (P110-118, Medical Statistics and CHISS Application) [17]. Statistical significance was defined as P value < 0.05.

## Results

There were no statistically significant differences of patients age and operating time between the open group and the laparoscopic one (P = 0.1694, 0.4244, respectively). There was no statistical constituent ratios difference of the unilateral and bilateral patent processus vaginalis between the two groups (P = 0.3577). The postoperative stay in hospital and the follow-up time for the laparoscopic group was significantly shorter than that of the open group (P = 0.0000, 0.0000, respectively) (Table 2). There were no postoperative complications for all the patients. No patient in the laparoscopic group was converted to open surgery. Ten patients were needed for the hand–eye coordination of this process.

## Discussion

There were no statistical significances of the patients age, operating time and side constituent ratio between the open group and the laparoscopic group. The postoperative stay in hospital of the laparoscopic group was significantly shorter than that of the open group. The following-up time of the laparoscopic group was shorter than that of the open group. There were no postoperative complications for all the patients. There was no conversion of the laparoscopic ones. From the above results, we can see that umbilical two-port laparoscopic percutaneous extraperitoneal closure of patent processus vaginalis was feasible and safe.

Comparing with single-hand manipulation, double-hand manipulation of the instruments was relatively easier for the operator to establish the laparoscopic depth perception [13]. In order to separate the peritoneum from the vas deferens conveniently, an assistant dissecting forceps was necessary for laparoscopic extraperitoneal closure of the internal ring. Umbilical two-port method of laparoscopic percutaneous extraperitoneal closure of the patent processus vaginalis balanced the coordination and cosmetic effects. During the whole process, the lens pole movements were parallel and synchronous with that of the dissecting forceps. This meant the operator and assistant pursued and coordinated a state of solo-like surgery [18–20]. This solo-like manipulation helped the operator to establish depth perception, which was very valuable for a novice. This synchronous and parallel movements of the umbilical two-port instruments made the operator felt that the lens pole was controlling by his or her own eventually. This umbilical instrument assignment and parellel movement not only overcame the possible instrumental bump, but also achieved the solo-like coordination of the instruments more easily due to a shorter distance between the two instruments (lens pole and dissecting forceps).

**Fig. 10:**
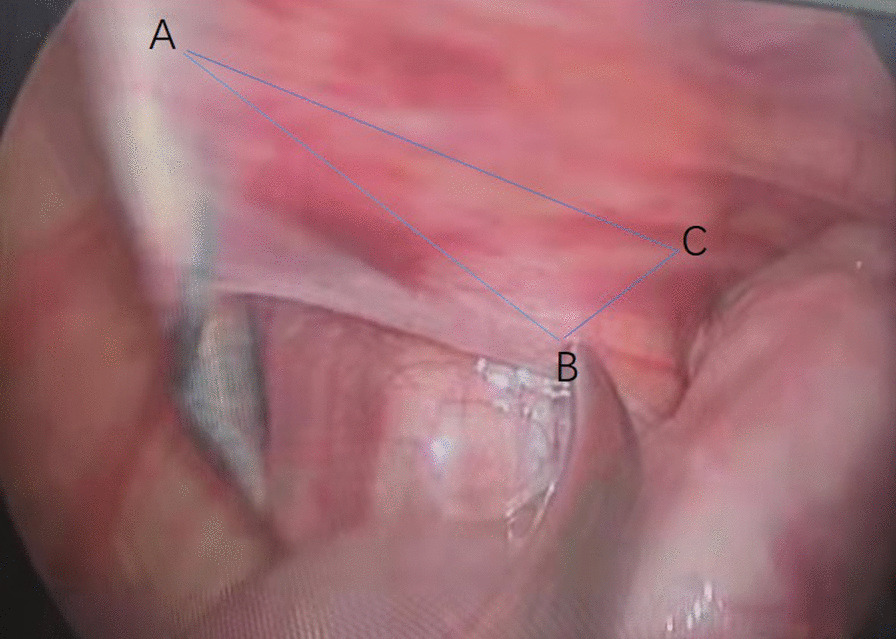
The imaginary manipulation plane for dissection of vas deferens and vessels. **A** was the needle end site, **B** was the forceps tip site and **C** was the protrude of vas deferens crossing the internal ring

Although the operator adopted double-hand manipulation of the instruments for orientation establishment, the laparoscopic perception was still two-dimensional for the novice at first. To establish a three-dimensional perception in a two-dimensional view, selecting a third anatomic protrude to imagine a manipulating plane in mind might be a good choice. The other two points to form the trianglar manipulating plane were the needle tip and the forceps tip for this process. The protrude where the vas deferens crossing over the internal ring was suitable to be selected as the third point. And an imagined triangle manipulating plane was formed (Fig. 10). Once the manipulating plane had been formed in the operator’s mind, the following surgical process could be completed in this imaged plane. Namely, the laparoscopic orientation perception absence was overcome through manipulation on this imagined plane. Similar studies were mentioned in the following articles: M. Wentink suggested that a clear visible endoscopic instrument shaft on the monitor facilitated hand–eye coordination [21]. Akio Kaito mentioned the concept of “triangulation” and “move the ground”. The operator and assistant exposed the operative field as triangle shape. And as the axis of surgical devices fixing, the dissection line should be adjusted by move the ground method [22]. Magdalena K. Chmarra suggested that movements during laparoscopic tasks were not performed via the shortest path. The retracting phase was important for the depth perception and the seeking phase was significantly different between experts and novices [23]. In this study, the authors established three-dimensional perception when 10 patients performance had been accomplished. During the whole laparoscopic process, we adopted gazing the view method, which was believed to use the visual information to plan and control tool movements [24–26]. More data should be accumulated to study the eye-hand coordination for this method in the future.

This self-made needle had a round dull shovel-like tip which endowed it a suitable sharpness for puncturing conveniently. No instrument damage occurred in this study. There was a central hole in the shovel which allowed a silk thread crossing through. This carrier lowered the requirement standard of the thread material for internal ring ligation, which made traditional silk thread ligation available in laparoscopic processus vaginalis closure. The disposable dissecting forceps was characteristic with a 30-degree bend, which was light and handy for manipulation.

There were no postoperative complications for all the patients. The postoperative stay in hospital for the laparoscopic group was significantly shorter than that of the open group. This quick postoperative recovery was another advantage for laparoscopic surgery. The remote effects of laparoscopic group should be continuously traced.

## Conclusions

Our preliminary experience suggested that umbilical two-port laparoscopic percutaneous extraperitoneal closure is safe and convenient for patent processus vaginalis treatment in boys. It has the advantage of incision-hiding and can be manipulated like a solo-like surgery.

## Data Availability

All data is contained within the manuscript and its additional files. The datasets used and analysed during the current study available from the corresponding author on reasonable request.

## Abbreviations

CHISS: Chinese High Intellectualized Statistical Software
